# Recurrent Invasive *Staphylococcus aureus* Infections in a Non‐Neutropenic Hemodialysis Patient With Myelodysplastic Syndrome: A Case Report

**DOI:** 10.1155/crin/1023646

**Published:** 2026-10-09

**Authors:** Keisuke Ono, Nobuhiro Ayuzawa, Takahisa Kawakami, Koichi Taniwaki, Shiori Arai, Aoi Kanematsu, Yuki Suhara, Mitsumasa Kishimoto, Yoshinori Komagata

**Affiliations:** ^1^ Department of Nephrology and Rheumatology Kyorin University School of Medicine Tokyo Japan kyorin-u.ac.jp

**Keywords:** hemodialysis, myelodysplastic syndrome, *Staphylococcus aureus*

## Abstract

**Introduction:**

Myelodysplastic syndromes (MDS) may be overlooked in patients undergoing hemodialysis (HD) because anemia is commonly attributed to renal anemia and treated with erythropoiesis‐stimulating agents. MDS is also associated with an increased risk of bacterial infections due to neutropenia and qualitative immune dysfunction. We report recurrent invasive methicillin‐susceptible *Staphylococcus aureus* (MSSA) infections in a patient undergoing HD with concomitant MDS.

**Case Presentation:**

The patient was a 76‐year‐old man undergoing HD for end‐stage kidney disease due to nephrosclerosis who was referred to our hospital because of persistent anemia despite high‐dose darbepoetin alfa therapy. He had a history of CHOP therapy for diffuse large B‐cell lymphoma, and his MDS was considered possibly therapy related. Bone marrow aspiration revealed trilineage dysplasia and 3.4% blasts without chromosomal abnormalities, leading to a diagnosis of MDS with multilineage dysplasia. Approximately 1 year later, he developed persistent fever. Blood cultures yielded MSSA, and imaging studies revealed septic pulmonary embolism and multiple cerebral infarctions. He improved after intravenous cefepime therapy. Twenty‐two months later, he developed fever and left shoulder pain. Contrast‐enhanced computed tomography revealed an abscess in the left subscapularis muscle, and MSSA was isolated from the drainage fluid after CT‐guided drainage. He improved with drainage and antibiotic therapy. In both episodes, the neutrophil count was preserved.

**Conclusion:**

This case illustrates recurrent invasive MSSA infections in a non‐neutropenic HD patient with possibly therapy‐related MDS. HD‐related factors should be carefully evaluated as common causes of recurrent *S. aureus* infection, and MDS may represent an additional, unproven host factor. MDS should be considered in ESA‐resistant anemia in HD patients, particularly in those with a history of cytotoxic chemotherapy.

## 1. Introduction

Myelodysplastic syndromes (MDSs) are hematologic disorders that occur predominantly in older adults and are characterized by clonal hematopoiesis, cytopenias including anemia, and dysplasia [1]. Anemia is also an extremely common clinical finding in patients undergoing hemodialysis (HD), and many patients are treated with erythropoiesis‐stimulating agents (ESAs) [2–4]. Although there are a few reports of MDS in patients undergoing HD, MDS may be overlooked in this population because anemia is commonly attributed to renal anemia and treated with ESAs [5].

MDS is known to be associated with an increased risk of bacterial infections, not only because of neutropenia but also because of granulocyte dysfunction [6]. Indeed, infections have been reported to account for a substantial proportion of deaths among patients with MDS [7]. Patients undergoing HD are also susceptible to infections [8], and they are at increased risk of bloodstream infections due to repeated puncture of vascular access sites and other factors [9, 10]. Therefore, patients undergoing HD who also have MDS may require careful evaluation for infectious complications.

As described above, reports of patients undergoing HD complicated by MDS are scarce, and reports focusing in detail on infectious events in such patients are, to the best of our knowledge, extremely limited. Here, we report a patient undergoing HD with MDS who developed recurrent methicillin‐susceptible *Staphylococcus aureus* (MSSA) infections despite the absence of apparent severe neutropenia and without receiving immunosuppressive therapy.

## 2. Case Presentation

A 74‐year‐old man was started on HD for end‐stage kidney disease due to nephrosclerosis. He received maintenance HD three times weekly via a native arteriovenous fistula (AVF) at the left elbow. The same AVF was used during both infectious episodes. He had a history of diffuse large B‐cell lymphoma at the age of 57 years, for which he received six cycles of cyclophosphamide, doxorubicin, vincristine, and prednisone (CHOP) therapy and achieved remission. Two years after the initiation of HD, at the age of 76 years, he was referred to our university hospital because anemia of approximately 8 g/dL persisted despite high‐dose ESA therapy with darbepoetin alfa 180 μg per week. The serum ferritin level was 42 ng/mL, with a calculated transferrin saturation of 66.7%. There was no evidence of active inflammation. Peripheral blood tests showed a neutrophil count of 1932/μL and a platelet count of 132,000/μL. Bone marrow aspiration revealed trilineage dysplasia and 3.4% blasts, with no chromosomal abnormalities. Based on these findings, he was diagnosed with MDS with multilineage dysplasia according to the WHO classification, IPSS Low risk, and IPSS‐R Intermediate risk (score 3). Given his prior exposure to CHOP therapy, the MDS was considered possibly therapy related. He was subsequently followed with a plan to provide red blood cell transfusions if his anemia progressed.

### 2.1. Episode 1

Approximately 1 year after the diagnosis of MDS, at the age of 77 years, the patient was referred and admitted to our hospital because of fever persisting for 10 days despite levofloxacin (LVFX) therapy (Day 1 of Episode 1). On admission, blood tests showed a markedly elevated C‐reactive protein (CRP) level of 30.02 mg/dL, whereas the neutrophil count was not decreased, at 16,262/μL. Blood tests performed at his HD clinic 9 days before presentation had also shown no decrease in the neutrophil count, at 2517/μL. Imaging studies revealed septic pulmonary embolism on computed tomography (CT) and multiple cerebral infarctions on brain magnetic resonance imaging (MRI) although the infectious focus was not evident at the time of admission. On admission, his body temperature was 37.3°C, blood pressure was 114/49 mmHg, pulse rate was 133 beats/min, respiratory rate was 16 breaths/min, and SpO_2_ was 95% on room air. He was alert. He met at least two systemic inflammatory response syndrome (SIRS) criteria, namely, tachycardia and leukocytosis.

Blood cultures collected on Day 1 from a vein in the right upper limb and the right femoral artery both yielded MSSA. No blood cultures were obtained from the AVF. The blood isolate was susceptible to oxacillin and cefazolin. Cefepime and cefoxitin were not included in the antimicrobial susceptibility testing panel. Intravenous cefepime was administered from Day 4 to Day 35. De‐escalation was not performed even after the susceptibility results became available because the possibility of mixed infection in the pulmonary lesions could not be completely excluded and because drug penetration into the central nervous system was considered in relation to the multiple cerebral infarctions. Multiple carious teeth were noted during hospitalization, but pulpal or periapical involvement could not be confirmed. No rash, petechiae, or purpura were observed, including around the AVF cannulation site on his left arm. The portal of entry remained unidentified. Transthoracic echocardiography (TTE) was performed, but no definite vegetation was observed. TEE could not be performed because of staffing and equipment constraints during the COVID‐19 pandemic. With antibiotic therapy, the patient’s clinical symptoms and CRP level improved steadily, and the inflammatory response remained negative after completion of antibiotic therapy (shown in Figure 1).

**FIGURE 1:**
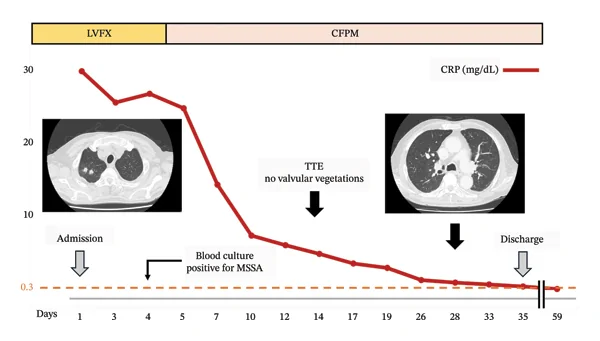
Clinical course of Episode 1. The patient developed MSSA bacteremia with septic pulmonary embolism and multiple cerebral infarctions. CRP levels decreased after treatment with cefepime, and transthoracic echocardiography showed no valvular vegetations. Abbreviations: CRP, C‐reactive protein; CFPM, cefepime; LVFX, levofloxacin; MSSA, methicillin‐susceptible *Staphylococcus aureus*; TTE, transthoracic echocardiography.

### 2.2. Episode 2

The patient remained free from recurrent infection for approximately 1 year and 9 months after Episode 1. Twenty‐two months later, he developed left shoulder pain and fever without any apparent history of trauma. Antibiotic therapy with cefditoren pivoxil was initiated at a local clinic. Blood tests performed during HD on the following day showed a marked inflammatory response, with a CRP level of 18.07 mg/dL, and he was referred and admitted to our hospital on the same day (Day 1 of Episode 2). At this time, the neutrophil count was also not decreased, at 2117/μL. Contrast‐enhanced CT revealed an abscess in the left subscapularis muscle, which was considered the infectious focus, although the abscess was too small to puncture at the time of admission. Blood cultures obtained on Day 1 were negative; however, this result should be interpreted with caution because cefditoren pivoxil had been administered before admission.

Ampicillin/sulbactam was started on the day of admission. However, the left shoulder pain worsened, and the CRP level remained elevated at 29.0 mg/dL on Day 8. Therefore, antimicrobial therapy was escalated to a combination of meropenem and vancomycin from Day 8. Repeat CT on Day 10 showed enlargement of the abscess, and CT‐guided percutaneous drainage was performed on Day 11. MSSA was detected in the drainage fluid culture, and the isolated strain was susceptible to oxacillin and cefazolin. The antibiotic regimen was switched to cefazolin on Day 15. The clinical symptoms, laboratory findings, and imaging findings improved, and intravenous cefazolin was switched to oral cephalexin on Day 26 and continued until Day 49. Shortly before this episode, the patient underwent caries removal and filling without tooth extraction. Pulpal necrosis, apical periodontitis, and periapical abscess were not documented. Examination again revealed no rash, including around the AVF cannulation site. Despite the temporal association with the dental procedure, the portal of entry could not be established. The inflammatory response remained negative after completion of antibiotic therapy (shown in Figure 2).

**FIGURE 2:**
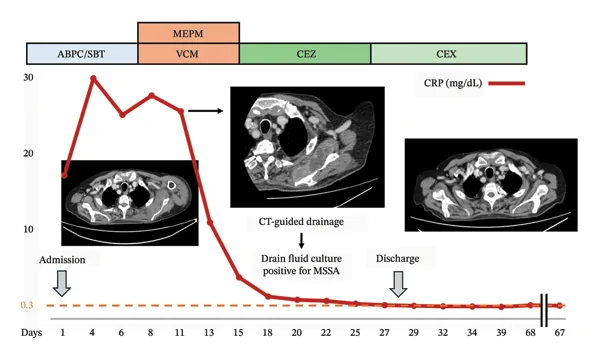
Clinical course of Episode 2. The patient developed an abscess in the left subscapularis muscle. MSSA was isolated from the drainage fluid after CT‐guided drainage, and CRP levels decreased after drainage and antibiotic therapy. Abbreviations: ABPC/SBT, ampicillin/sulbactam; CEZ, cefazolin; CEX, cephalexin; CRP, C‐reactive protein; CT, computed tomography; MEPM, meropenem; MSSA, methicillin‐susceptible *Staphylococcus aureus*; VCM, vancomycin.

## 3. Discussion

We report a non‐neutropenic patient undergoing maintenance HD who was diagnosed with MDS after evaluation for ESA‐resistant anemia and subsequently developed two episodes of invasive MSSA infection. Because the patient had previously received CHOP therapy for diffuse large B‐cell lymphoma, the MDS was considered possibly therapy related. This case provides an educational example of recurrent invasive MSSA infection in an HD patient with possibly therapy‐related MDS and highlights the need for cautious interpretation of infection risk in such patients.

Anemia is a common clinical finding in patients undergoing HD [3] and is a target for therapeutic intervention because it is associated with prognosis [11]. The mainstay of treatment is ESA therapy, and many patients undergoing HD receive ESAs for the treatment of anemia [4]. Although anemia improves with ESA therapy in many patients, approximately 5%–10% of patients, depending on the definition used, do not achieve an adequate hematologic response even with high‐dose ESAs. Various factors, including inflammation, iron deficiency, uremia, malnutrition, secondary hyperparathyroidism, occult bleeding, and vitamin B12 or folate deficiency, have been implicated in ESA hyporesponsiveness [12]. Large‐scale epidemiologic data on the coexistence of MDS in patients undergoing HD are limited, and MDS is not generally recognized as being particularly frequent in this population. However, a cohort study of 20 maintenance HD patients with MDS reported that lower‐risk MDS may present only with anemia and that patients diagnosed after the initiation of dialysis often already had cytopenias, especially thrombocytopenia, at the time of diagnosis [5]. These findings suggest that MDS may be difficult to detect in patients undergoing HD. In the present case, persistent anemia of approximately 8 g/dL despite high‐dose darbepoetin alfa therapy after the initiation of HD led to bone marrow evaluation and the diagnosis of MDS. Therefore, when ESA‐resistant anemia is observed in patients undergoing HD, hematologic disorders including MDS should be considered in addition to common causes related to renal anemia, particularly in patients with prior exposure to cytotoxic chemotherapy.

Infections are important complications in patients with MDS. Although neutropenia is a major factor contributing to infection risk, the risk of infection may not be fully explained by the peripheral neutrophil count alone. Neutropenia has been reported in approximately 50% of newly diagnosed patients with MDS, including 70%–80% of those with higher‐risk MDS and 15%–20% of those with lower‐risk MDS [6]. In a recent large‐scale analysis using the European MDS Registry, a low absolute neutrophil count was independently associated with infections in general, whereas it was not independently associated with infection‐related death among patients who developed infections [13]. In MDS, qualitative functional abnormalities of neutrophils, including impaired chemotaxis, adhesion, phagocytosis, reactive oxygen species production and bactericidal activity, have been reported, and these abnormalities may impair host defense even in patients with preserved neutrophil counts [6]. Indeed, neutrophils from patients with MDS have been reported to have reduced bactericidal and fungicidal activity compared with neutrophils from healthy individuals [14]. In addition, a cohort study of patients with lower‐risk MDS reported that infections were the most common cause of death and accounted for approximately one‐third of deaths [7]. The European MDS Registry also showed that infections were a major cause of death within the first year after diagnosis among patients with lower‐risk MDS [13]. In the present case, peripheral neutrophil counts were preserved during both infectious episodes, making susceptibility due to severe neutropenia unlikely. However, neutrophil function tests were not performed, and MDS‐associated qualitative immune dysfunction could not be directly demonstrated. Thus, MDS‐related immune dysfunction should be regarded only as a possible, unproven contributor to the development and severity of MSSA infections in this patient. This case suggests that preserved neutrophil counts do not necessarily exclude impaired host defense in patients with MDS.

Patients undergoing HD are at high risk for bloodstream infections because of uremia‐associated immune dysfunction, repeated cannulation of vascular access sites, disruption of the skin barrier, and frequent exposure to healthcare settings [8, 9]. In particular, *S*. *aureus* is an important causative pathogen of bloodstream infections in patients undergoing HD, and vascular access can serve as a major portal of entry [9, 10, 15]. *S*. *aureus* bacteremia is prone to complications such as infective endocarditis and metastatic infectious foci, including vertebral osteomyelitis, abscess formation, and septic embolism [16]. In the present case, Episode 1 was characterized by MSSA bacteremia complicated by septic pulmonary embolism and multiple cerebral infarctions. Parsi et al. reported MSSA bacteremia and tricuspid valve infective endocarditis in a patient receiving maintenance HD without apparent signs of AVF infection [17]. This report highlights the importance of evaluating for infective endocarditis even in the absence of local signs of AVF infection. In our patient, TTE showed no definite vegetation during Episode 1, but TEE was not performed, limiting the evaluation for infective endocarditis. Therefore, occult infective endocarditis could not be completely excluded. In Episode 2, although blood cultures were negative, MSSA was detected in the drainage fluid from the left subscapularis muscle abscess. The negative blood cultures in Episode 2 should be interpreted with caution because cefditoren pivoxil had been administered before admission. A definitive portal of entry was not identified in either episode. Therefore, when patients undergoing HD with concomitant MDS present with fever or focal pain, clinicians should first evaluate common causes of *S. aureus* infection by obtaining repeated blood cultures, carefully inspecting the entire skin surface and vascular access site, evaluating for dental infection, performing echocardiography when bacteremia is suspected, and searching for deep infectious foci using CT or MRI.

In the patient receiving maintenance HD reported by Skrypnyk et al., infection of a tunneled right internal jugular vein catheter spread contiguously to the sternocleidomastoid muscle and sternoclavicular joint, resulting in muscle phlegmon and septic arthritis, accompanied by erosion of the sternal end of the clavicle, septic pulmonary emboli, and a hemorrhagic petechial rash [18]. The patient presented with fever and right‐sided neck pain and swelling, with a CRP level of 8.9 mg/dL and Hb levels of 5.7–6.2 g/dL. Our patient likewise developed invasive MSSA infection during maintenance HD, with septic pulmonary embolic lesions and anemia during the first episode. However, he received HD through an AVF, and the portal of entry remained unidentified. Our patient also exhibited no rash, petechiae, or purpura during either episode. The previously reported patient improved following catheter removal, surgical drainage, and a 6‐week course of cefazolin [18]. Both cases illustrate that MSSA infection in patients receiving HD can involve deep tissues and the lungs. The distinctive features of our case were concomitant MDS and temporally separated episodes of invasive infection at different sites in the absence of neutropenia.

This report has several limitations. First, neutrophil function tests were not performed; therefore, qualitative immune dysfunction associated with MDS could not be directly demonstrated. Second, the definitive portal of entry of MSSA was not identified in either episode. Third, because HD itself is a strong risk factor for recurrent *S*. *aureus* infection, the contribution of MDS to recurrent MSSA infections in this patient could not be clearly distinguished from the infection risk associated with HD [19]. Fourth, because this is a single case report, a causal relationship between MDS and recurrent MSSA infection cannot be established. Nevertheless, the recurrence of invasive MSSA infections with deep or disseminated infectious manifestations in a non‐neutropenic patient undergoing HD with possibly therapy‐related MDS is clinically important. In patients undergoing HD who have ESA‐resistant anemia, MDS should be considered in the differential diagnosis, particularly when there is a history of cytotoxic chemotherapy. Furthermore, when HD patients with MDS develop fever or focal pain, common sources of *S. aureus* infection should be carefully evaluated, while MDS should be regarded only as a possible additional host factor.

## Funding

This study was not supported by any sponsor or funder.

## Ethics Statement

Ethics approval was not required for this case report in accordance with institutional requirements.

## Consent

Written informed consent was obtained from the patient for publication of the details of this medical case and any accompanying images.

## Conflicts of Interest

The authors declare no conflicts of interest.

## Supporting Information

Additional supporting information can be found online in the Supporting Information section.

## Supporting information


**Supporting Information** Care‐checklist_CN.

## Data Availability

The data underlying this case report are not publicly available due to privacy and ethical restrictions. Additional anonymized information may be available from the corresponding author upon reasonable request and with appropriate institutional approval.
